# Novel strategies lead to pre-elimination of malaria in previously high-risk areas in Suriname, South America

**DOI:** 10.1186/1475-2875-11-10

**Published:** 2012-01-09

**Authors:** Hélène Hiwat, Loretta S Hardjopawiro, Willem Takken, Leopoldo Villegas

**Affiliations:** 1 Malaria Programme, Ministry of Health Suriname, c/o Bureau of Public Health Suriname, Rode Kruislaan 13, Paramaribo, Suriname; 2 Laboratory of Entomology, Wageningen University and Research Centre, PO Box 8031, 6700, EH Wageningen, The Netherlands; 3 International Public Health Advisor, Dieterstraat 16, Paramaribo, Suriname

**Keywords:** Malaria control, Suriname, Insecticide-treated nets, Pre-elimination

## Abstract

**Background:**

Suriname was a high malaria risk country before the introduction of a new five-year malaria control program in 2005, the Medical Mission Malaria Programme (MM-MP). Malaria was endemic in the forested interior, where especially the stabile village communities were affected.

**Case description:**

The interventions of the MM-MP included new strategies for prevention, vector control, case management, behavioral change communication (BCC)/information, education and communication (IEC), and strengthening of the health system (surveillance, monitoring and evaluation and epidemic detection system). After a slow first year with non-satisfying scores for the performance indicators, the MM-MP truly engaged in its intervention activities in 2006 and kept its performance up until the end of 2009. A total of 69,994 long-lasting insecticide-treated nets were distributed and more than 15,000 nets re-impregnated. In high-risk areas, this was complemented with residual spraying of insecticides. Over 10,000 people were screened with active case detection in outbreak and high-risk areas. Additional notification points were established and the national health system was strengthened.

**Discussion and evaluation:**

In the current paper, the MM-MP is evaluated both on account of the targets established within the programme and on account of its impact on the malaria situation in Suriname. Malaria vector populations, monitored in sentinel sites, collapsed after 2006 and concurrently the number of national malaria cases decreased from 8,618 in 2005 to 1,509 in 2009. Malaria transmission risk shifted from the stabile village communities to the mobile gold mining communities, especially those along the French Guiana border.

**Conclusions:**

The novel strategies for malaria control introduced in Suriname within the MM-MP have led to a significant decrease in the national malaria burden. The challenge is to further reduce malaria using the available strategies as appropriate in the affected areas and populations. Elimination of malaria in the country will require a thorough understanding of transmission dynamics and a dedicated investment in key effective interventions.

## Background

The Amazon basin harbors 95% of the total malaria burden in the region and 98% of the *Plasmodium falciparum *infections of the Americas [1,2]. The Guyanan Shield area (Suriname, Guyana and French Guiana) is responsible for the highest numbers and concentration of *P. falciparum *cases in the Americas [1]. Malaria in Suriname is historically divided in two endemic areas; the coastal belt and the interior [3-6]. The coastal area was free of malaria by 1968 as a result of a DDT spraying campaign. In the interior spraying was done twice a year, but spray coverage was generally below 40% due to refusals of, and communication problems with the local population and malaria elimination was not achieved [4,5,7]. During the 1990s, a significant increase in malaria incidence in Suriname was observed. This increase was related to the improvement of malaria diagnosis, the increase of anti-malarial drug resistance to treatment of falciparum malaria (4-aminoquinolines [4,8]) and population movements due to internal conflicts. Suriname was considered as one of the countries with the highest annual parasite index (API) of malaria in the Americas [1].

Artemisinin-based combination therapy (ACT) was introduced in late 2003. A moderate decline in the number of cases was observed after the nationwide implementation of ACT as first-line treatment for uncomplicated *P. falciparum *infections in 2004 and 2005.

The Global Fund to fight AIDS, Tuberculosis and Malaria (GFATM), established in 2002 as a new mechanism to finance a rapid international effort to control the three diseases, approved a malaria proposal submitted by the Surinamese Country Coordinating Mechanism (CCM) in round 4 (R4) [9]. A five-year grant was provided to the Medical Mission (MM), a local government-supported non-governmental organization as principal recipient for a malaria programme. It was termed Medical Mission Malaria Programme (MM-MP) and aimed to reduce the transmission of malaria in high-risk communities in the interior of Suriname. The interventions of the MM-MP were in line with the Roll Back Malaria Partnership strategy [1] including activities in prevention, case management, behavioral change communication (BCC)/information, education and communication (IEC), and strengthening of the health system (surveillance, monitoring and evaluation and epidemic detection system). In this paper, the achievements of the MM-MP with regard to the programmatic performance indicators are described and the impact of the MM-MP on malaria incidence and transmission in Suriname is evaluated.

## Case description

### Study area

**Figure 1:**
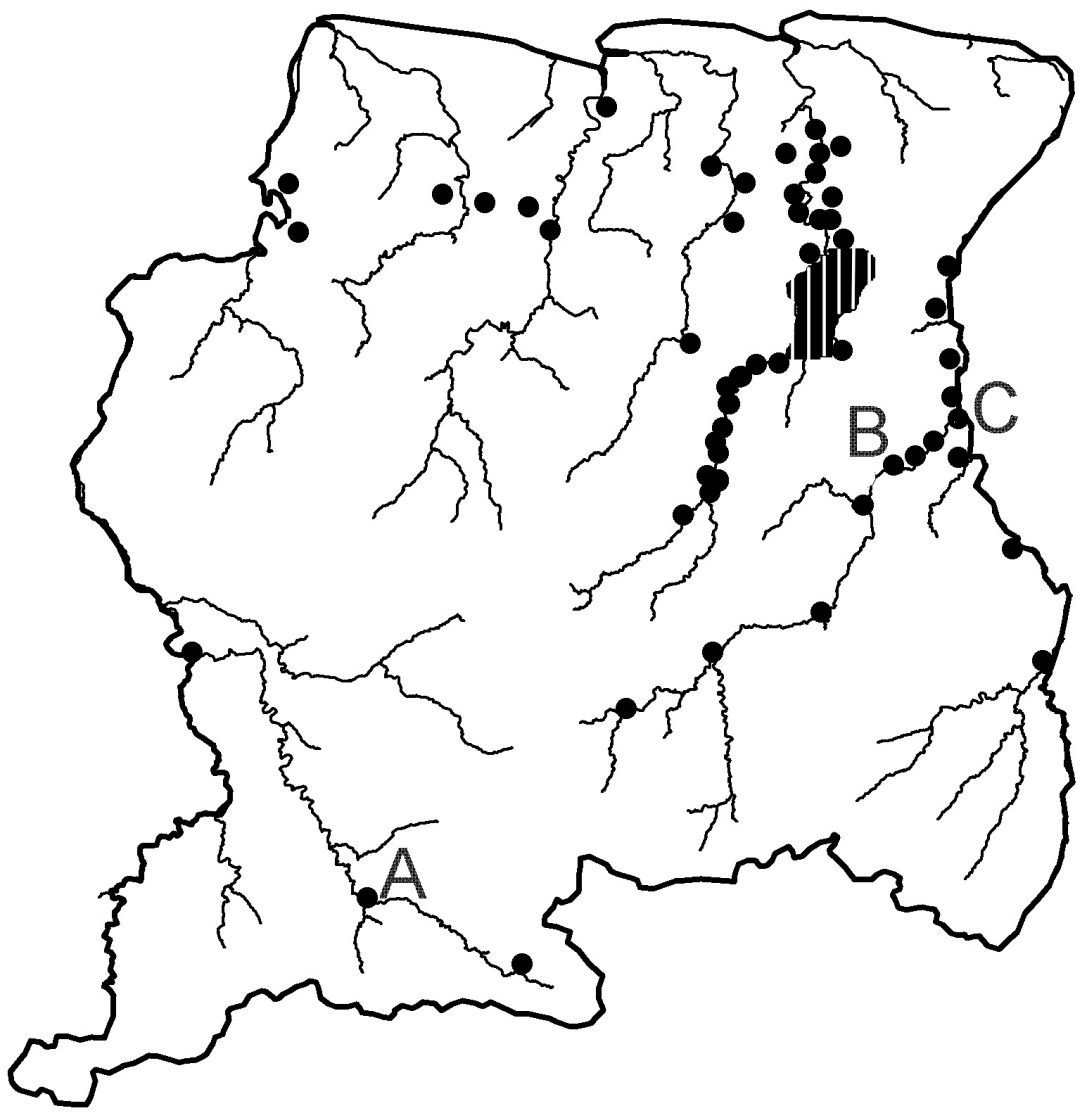
**Map of Suriname, showing the Medical Mission clinics (black dots) and the sentinel sites for entomological surveillance (A: Kwamalasamutu, B: Drietabiki, C: Stoelmans-island)**.

Suriname is part of the northern range of the Amazon forest, located between 2° and 6° latitude North and 54° and 58° longitude West along the North Coast of South America. The coastal plain is separated from the interior by a so-called savannah belt: poor agricultural land, consisting of a white-sand formation covered with shrubs. The 2004 census showed that Suriname had a population of almost 500,000 people, with 49.3% living in and around the capital, and only 9.8% living in the tropical rainforest of the interior [10]. The population of the interior consists of Amerindians and Maroons, living in tribal communities along the main rivers, and a number of immigrants who live and work in small-scale gold mines in the forest. A limited road system exists, which does not extend beyond the Van Blommensteyn Lake, about 150 kilometers into the interior (Figure 1). As a result, transportation to the villages is mostly by boat or plane. The population in the interior varies due to movements during the year and is estimated to be about 48,000 people with additional 15,000 mobile gold miners. The total population at risk for malaria in the country is 63,000 inhabitants. Human migration from and to Suriname is common, particularly along the French Guiana border.

Environmental conditions favor malaria transmission in Suriname. The climate is hot and humid, with an average temp of 27°C and annual relative humidity of around 80%. Four seasons are identified: the major rainy season from mid-April to mid-August; the major dry season from mid-August to November; the minor rainy season from December to January; and the minor dry season from February to mid-April.

Three human malaria parasite species are present in Suriname: *P. falciparum, Plasmodium vivax *and *Plasmodium malariae*. The majority of malaria cases reported from the interior are due to *P. falciparum *among the Maroons (descendants of African slaves), many of which live in high malaria risk areas, and generally showing a natural resistance against *P. vivax *infections. The Amerindians, the second largest ethnic group in the interior, and Brazilian gold miners ("*garimpeiros*") proved susceptible to all three malaria species. Mixed infections seldom occur (less than 2%). *Anopheles darlingi *is the primary vector. This species represents over 90% of the anophelines collected in previous entomological surveys [11-14]. Rozendaal [6] established that peaks in biting densities of *An. darlingi *correlated well with periods of (i) high water levels in the long rainy season, (ii) low water levels in the long dry season, and (iii) abundant rainfall in the short rainy season.

The Ministry of Health in Suriname (MoH) is responsible for all actions related to disease prevention and control. All but one hospital are located in the capital, Paramaribo. The Regional Health Service (RGD) provides primary health care in the Coastal areas. MM is responsible for the primary health care in the interior with 56 permanent health centers strategically located, which serve as diagnostic and treatment points for the people living and working in this area (Figure 1). The health centers are distributed over five medical regions. Each region has a non-resident medical doctor who supervises and assists local health-care workers through regular visits and radio contact. The health-care workers are able to provide diagnosis and first line treatment for the most common acute diseases; severe cases are referred to the Diakonessen hospital in the capital. Confirmed malaria cases are reported weekly to the MM headquarters via radio aiming to detect epidemics. Malaria reports are sent monthly to MM headquarters in Paramaribo by the MM health workers. For malaria diagnosis 17 of the 56 health centers have trained microscopists, the remaining health centers rely on the use of rapid diagnostic tests (RDTs) or so-called "dipsticks", which can detect antigens of malaria parasites in the blood of infected individuals. RDT results are cross-checked by microscopic analyses of blood slides at the MM Headquarter in Paramaribo [15]. Suriname participates in the Amazon Network for Surveillance of Anti-malarial Drug Resistance-Amazon Malaria Initiative (RAVREDA-AMI) and as such takes part in an evaluation of the use of RDTs as an alternative for microscopy [16] and as a quality control of microscopic diagnosis.

For technical advice on malaria prevention, diagnosis, treatment and vector control, the MoH relies on the national malaria board, which is a national advisory technical group. The Bureau of Public Health (BOG) is responsible for national malaria control. Data on malaria morbidity and mortality is routinely obtained from health care and malaria providers in the country and centralized at BOG. A malaria information system, which includes a database with weekly recordings of malaria data from the individual health service providers, was set up in 1955. It has been continuously upgraded since then, and represents the backbone of the national surveillance.

### Performance framework and monitoring progress

The MM-MP was a performance-based programme funded by the GFATM. Each GFATM grant consists of two phases, which are continuously monitored with an agreed-upon performance framework. Quarterly audits of grants are usually conducted during the first years, and after that semi-annually. The performance framework is the formal statement of the performance expected over the lifetime of the grant as signed in the grant agreement. It contains a summary of key indicators and targets, which are used to measure i) programme outputs and coverage on a routine basis and ii) programme outcomes and impact [17]. The performance rating of a grant programme is based on i) the overall progress achieved against time-bound targets for key output indicators; and ii) an assessment of management performance (in the areas of monitoring and evaluation (M&E) financial and programme management, and procurements of pharmaceuticals and health products). The programme is graded an "A1" or "A2" [exceeding (> 100%) or meeting performance expectations (90-100%)], a "B1" (adequate performance 60-89%), a "B2" (inadequate performance but with potential demonstrated, 30-59%) or a "C" (unacceptably poor performance, < 30%) [18]. Evaluation of the MM-MP is done in 23 periods based on the achievements in the performance framework.

### MM-MP Interventions

#### Vector control

Sentinel sites for entomological surveillance were established in three key locations: Kwamalasamutu (near the Brazilian border), Jamaica (Upper Marwoijne River, along the border with French Guiana) and Dritabiki (along the Tapanahony River) (Figure 1). Simultaneous all-night indoor and outdoor collections were done during surveys of 3 nights, each quarter of the year. Mosquitoes were collected with the human landing collection method (approved by the Ministry of Health of Suriname under project no. VG2006-006). All anophelines collected were tested on *Plasmodium *infection with the sandwich enzyme linked immunosorbent assay (ELISA) detection according to Wirtz *et al*.[19]. In 2006 and 2007, this was done with 96 man-hours per night, from 2008 onwards this was reduced to 24 man-hours per night.

Over the years, conventional bed nets for prevention of malaria had been distributed in Suriname, with funds from national and international donors. A programme of manufacturing by, and marketing through, women group organizations at the local level was established in 1997. Since 2003 these locally-produced bed nets have been impregnated on site with pyrethroids. The MM-MP introduced long-lasting insecticide-treated nets (LLINs) (PermaNet 2.0^®^, Vestergaard-Frandsen, Switzerland), which were distributed for free to people living in communities in the interior. The target was to distribute 73,000 ITNs to the communities of the interior (Table 1 performance indicator 2). One LLIN was available for each person and two LLINs if the person was a pregnant woman. Impregnation kits containing deltamethrin WT 25% (KO-Tab 123^®^, Bayer Pty., Ltd) were used for impregnation and re-impregnation of nets since 2006 (Table 1 performance indicator 8). Insecticide-treated nets (ITNs), including LLINs, were re-impregnated in high-risk areas. Different means of transportation (i.e. cars, boats, airplanes, all terrain vehicles) and stakeholders' participation were used for the implementation of all interventions.

**Table 1:** Comparison of performance indicators for the MM-MP: baseline vs.

Performance indicators	Baseline	Final Results		%	Δ
	
	Value [2004]	Target	Achieved	Achieved	Change^
1) No. of service deliverers trained in the use of LLINs and prompt, effective, anti-malarial treatment^#^	0	2,333	2,184	93.6	+2,184

2) No. of free nets distributed to the indigenous populations and gold miners	0	73,000	69,994	95.8	+69,994

3) No. (and %) of uncomplicated malaria cases receiving correct diagnosis and treatment ^@^	(60%)	74	112	151	+112

4) No. (and %) of health clinics in the interior with no reported stock-outs of anti-malarial drugs	0	56	2	3.6	+2

5) No. (and %) of severe malaria cases receiving correct diagnosis and treatment	162	8	9	112.5	-153

6) No. of fixed/mobile malaria service delivery points established for gold miners.	0	43	31	72.1	+31

7) No. of health facilities reporting information on a regular basis using standardized information systems	0	56	56	100	+56

8) No. of ITNs impregnated and/or re-impregnated	0	600	327	54.5	+327

9) No. of health and community personnel trained in malaria control activities^&^	0	118	110	93.2	+110

10) No. of communities reached by the malaria media awareness campaign	0	140	58	41.4	+58

11) No. of people living in the interior reached through BCC activities	0	3170	610	19.2	+610

final results

^#^including health workers and community personnel

^@^including children under 5

^&^including epidemiology, entomology, residual spraying, etc.

^^^compared to baseline value

Indoor Residual Spraying (IRS) with alpha-cypermethrin (Fendona^®^, BASF) was conducted only along the Upper Marowijne and Tapanahony Rivers areas, which were the areas with the highest API in the country. Local men and women were trained in IRS and recruited in an effort to reach high coverage and maximized community participation.

#### Case management

Among the performance indicators were the number and percentage of malaria cases and the number and percentage of severe malaria cases receiving correct diagnosis and treatment (Table 1 performance indicator 3 and 5). Paracheck-Pf^® ^(Orchid Biomedical Systems) has been routinely used as a RDT for diagnosis of falciparum malaria in MM health post without microscopists. Since 2005, Pf/Pan specific tests were introduced as national policy in the country (BinaxNOW^® ^Malaria, Inverness Medical Innovations, Inc.). All health service providers received refreshment training in malaria diagnosis and treatment (Table 1, performance indicator 1). A pilot outreach activity started late in 2005 aiming to provide malaria services to populations without access to health services in remote areas, especially in gold mining areas, via the introduction of Malaria Service Deliverers (MSDs) (Table 1 performance indicator 6). MSDs are fixed/mobile local persons trained in diagnosis and treatment of malaria who provide free services in remote areas. MSDs receive supplies and supervision by the malaria control programme. MSD points were established as a pilot in key gold mines areas where malaria cases where previously reported. In 2007, a fixed MSD was established in northern Paramaribo to provide malaria service to the large Brazilian (*"garimpeiro"*) community living there. This is the only MSD point with trained microscopists. Northern Paramaribo is the commercial centre for purchasing mining equipment and supplies and to trade off gold. Mine workers move frequently between northern Paramaribo and the mining fields in the interior.

An aggressive active case detection (ACD) campaign was initiated in 2006 in the gold mining areas where malaria is endemic. ACD was also performed during confirmed malaria outbreaks. All malaria service providers completed training in routine malaria surveillance and reporting mechanisms (Table 1 performance indicators 1 and 9).

#### Behavioural change communication (BCC) and information, education and communication (IEC)

The number of communities and the number of people reached by the BCC activities are part of the performance indicators (Table 1 performance indicators 10 and 11). A media awareness campaign was launched in 2006. It comprised of mass communication channels to increase the knowledge of malaria control and prevention. Several TV and radio spots, newspapers, flyers, and posters were distributed widely in the coastal areas and points of entry to Paramaribo. Posters, folders and videos (DVDs) were provided in different languages to communities in the interior by a social scientist. Several training sessions with health staff, community leaders and traditional healers in malaria prevention and control activities were conducted.

#### Intelligent surveillance: detection and response to epidemics

The epidemic detection and response system was strengthened (Table 1 performance indicator 7). Endemic channels were built using weekly malaria reports per each health/malaria reporting unit and standard operation procedures for epidemic containment were developed. Epidemic thresholds were established depending on local situations. In general the threshold for villages was set on three malaria cases per week, but if a village had been malaria free for a significant period of time even one case (as a result of local transmission) was considered an outbreak.

Malaria information is centralized in two databases; one at the MM headquarters and one at the anti-malaria campaign (AMC) of BOG. AMC collected all malaria data from blood bank, hospitals and laboratories in Paramaribo.

Quality control of slides analysed in the MM clinics in the interior was done at the MM headquarters in Paramaribo. Quality control of the RDT results from the MSDs was done by slide analysis (slides taken simultaneous with RDT tests) at the fixed MSD clinic in northern Paramaribo. Quality control of the fixed MSD clinic was done by regular supervised blind re-reading of slides by the available microscopists in this clinic (re-reading 100% of the positive slides and 10% of the negative slides).

#### Monitoring and evaluation (M&E)

The MM-MP standardized the data collection tools in the country. Case administration and reporting was improved by introduction of a new national format for case reporting. All health workers received training in the use of this new national format, which was piloted in 2007 and implemented in 2009. The malaria M&E system for the MM-MP was developed initially in 2006, refined in 2008 and was updated in 2010 became the national multi-sectoral M&E plan. An integrated database with reporting system was developed in 2009.

### Programme assessment

Malaria-related data i.e., outpatient and hospital admissions, were collected from notification units and triangulated with the BOG central database. Only confirmed cases were included in the malaria surveillance system. Information on anti-malarial drugs stock was collected monthly via radio (Table 1 performance indicator 4). Programmatic indicators were assessed using data reported by the MM-MP M&E unit. MM-MP databases on entomological surveillance and IRS and were reviewed. Pearson's chi square tests and Student tests were used to compare two percentages or two means respectively (α = 0.05) (calculated with SPSS^® ^version 17.0 (IBM Corporation, Somers NY, USA)). Δ change (per each performance indicator) was calculated comparing the baseline value of 2004 with final result achieved in the MM-MP. Changes of impact/outcome indicators were used to assess project overall achievements and its implications in public health. Programmatic and financial information were obtained from GFATM web site (grant portfolio SUR-404-G02-M) [11].

### Assessment results

The MM-MP began in February 2005 and ended in October 2010. Performance reports were done quarterly between February 2005 and January 2009 and semi-annually thereafter. There were 19 performance reports during the duration of the project.

### Interventions

For practical reasons interventions are grouped by strategic areas: vector control (including IRS, LLNs, re-/impregnation of nets and entomological surveillance); case management (diagnosis and treatment); BCC/IEC (mass media, outreach programme) and Surveillance, Monitoring and Evaluation (including epidemic detection, passive and active case surveillance, mobile/fixed malaria service deliverers, M&E).

### Vector control

#### Entomological surveillance

Entomology surveys were performed in Drietabiki (372 human-nights (hn)), in Jamaica (near Stoelmans-island) (372 hn) and in Kwamalasamutu (372 hn) between January 2006 and April 2010. The majority of anophelines collected during the entomological surveys of 2006 (5185 man-hours (mh)), 2007 (6048 mh), 2008 (864 mh), 2009 (864 mh) and 2010 (216 mh, until April) were *An. darlingi*, mostly collected in Drietabiki and Jamaica. Other anophelines collected were *Anopheles nuneztovari*, *Anopheles oswaldoi, Anopheles albimanus *and *Anopheles intermedius*. Biting by *An. darlingi *occurred during night time and showed a peak between 01.00 and 02.00 hr. at Jamaica, while at Drietabiki the peak was towards the early morning hours [20]. In all three sites the *An. darlingi *population showed a sharp decrease from 2006 onwards. *Anopheles darlingi *has not been collected in any of the three sentinel sites from 2008 onwards. Out of 683 anophelines tested for malaria infection during these years, two females were found infected with *Plasmodium falciparum*. Both specimens were collected in 2006, one originated from Jamaica and the other specimen from Drietabiki. Combining these data with the human biting rate found in these two sentinel sites, this results in an entomological inoculation rate (EIR) of 0.8 infected bites per month in Jamaica and 1.7 infected bites per month in Drietabiki, for the specified months in 2006. Yearly EIR would be very low as a result of the low population densities.

#### ITNs

LLINs distribution began early April 2006 and was temporary interrupted (for 2 months) due to extensive flooding of the rivers in May 2006 following unusually heavy rains. 55,100 LLINs - were distributed between 2006 and 2007 covering almost all stable communities in the interior. In 2008 and 2009, an additional 14,508 and 386 LLINs were distributed respectively to replace used ones in high risk areas and to supply small communities that had not previously received them. A total of 69,994 LLINs were distributed during the MM-MP. A monitoring and evaluation bed net survey conducted in 2007 indicated that 83% of the people were sleeping under a net (MM-MP Multiple Indicator Survey report 2007). There were nine gold mining communities (mobile communities) included in the bed net distribution of 2008, which received 1,212 LLINs.

#### Re-impregnation of nets

Training on the use of KO-Tab^® ^123 and local impregnation activities were executed in 332 communities. A total of 15,023 nets (conventional/LLINs) were re-impregnated between 2007 and 2009 and targets were achieved during most periods (Table 1, performance indicator 8). However, during the last year of the project, only 327 nets were re-impregnated which represented an achievement of 54.5% of the established target of 600 nets in 2010. This was most likely due to a decrease in the number of technical and field personnel employed by the MM-MP during the final year.

#### IRS

Two rounds of IRS were carried out in 2006 (June-August and September-November) only in communities along the Tapanahony- and Upper Marowijne areas, the highest malaria API areas in the country. These communities had also received ITNs. An overall coverage of 71% was achieved, ranging from 25% to 93%. In 3672 houses 4280 rooms were sprayed; 147 refusals were reported.

### Case management

The expertise of existing microscopy personnel was upgraded with 12 new microscopists trained and 19 working microscopists re-trained by qualified skilled professionals (Table 1 performance indicator 1). Only two out of the 56 MM health facilities (3.6%) reported no stock-out of any antimalarial drug at the end of the project and this was evident only at the end of the MM-MP (Table 1 performance indicator 4). Anti-malarial stocks were at 100% between January 2006 and October 2009. Primaquine, a drug used against vivax and falciparum malaria was absent from most health services from November 2009 till March 2010 due to procurements delays.

#### MSD

A total of 31 local workers (from target camps or communities) were selected as malaria service deliverer, achieving a 72.1% of the target established at the end of the project. The number of autochthonous malaria cases diagnosed among all MSD increased significantly from 500 in 2006 to 651 in 2009 (*p *= 0.021). The fixed MSD in the capital diagnosed 7% (122/1819), 16% (248/1597) and 19% (292/1509) of the total number of malaria cases in the country in 2007, 2008 and 2009, respectively (*p *< 0.17).

#### ACD

Thirty-four surveys were performed as active case detection in five areas (9 in 2006; 6 in 2007; 16 in 2008 and 3 in 2009). Overall, 10,702 people were screened for malaria with prevalence rates ranging from 0 to 60%. Malaria cases were diagnosed among 265 persons including 126 *P. falciparum*, 117 *P. vivax*, 20 *P. malariae *and two mixed infections. Higher prevalence rates were observed among people screened in gold mining areas. In 2010, ACD activities were taken over by a new malaria project under the coordination of the MoH. All infected people were treated according to the national treatment protocol.

#### Behavioural change communication (BCC) and information, education and communication (IEC)

Intensive awareness campaigns were initiated in 2006 and training sessions with the local population were conducted, especially with women group organizations. Women group organizations were targeted for education messages in malaria control and prevention, including the use and washing of LLINs, the use of insecticide kits for nets and prompt malaria diagnosis and treatment. At the end of the MM-MP¸ 58 communities and 610 people participated in the outreach media awareness campaign (Table 1 performance indicator 10, 41.4% achieved) and BCC (Table 1 performance indicator 11, 19.2% achieved) activities.

### Surveillance, monitoring and evaluation

**Figure 2:**
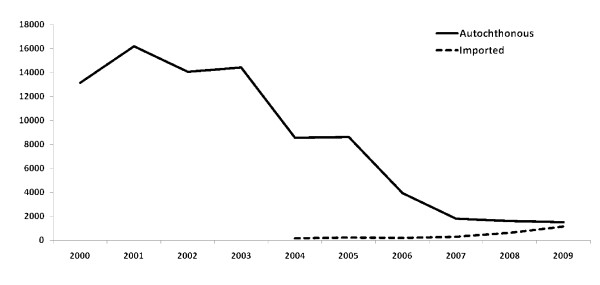
**Autochthonous and imported malaria cases in Suriname between 2000 and 2009**.

A total of 2,184 health service deliverers were trained in the use of LLINs and prompt effective anti-malarial treatment at the community level. All health services performing malaria diagnosis (108 centers nationwide) received training in RDTs and in the updated treatment protocol. Between 2005 and 2009, 156,878 blood samples were taken for malaria diagnosis with an annual average of 26,278 slides taken and 19,962 confirmed malaria cases diagnosed over the 5 years. While the number of autochthonous cases decreased, the number of imported malaria cases increased significantly (Figure 2), which resulted in a proportional increase from 3% in 2005 to 43.0% in 2009 (*p *< 0.001; Table 2). Most imported cases were diagnosed by the fixed MSD in the capital (Table 2) and by mobile MSDs in mining areas. The malaria passive surveillance system reported 17,463 cases over the 5 years of the project. The total number of autochthonous malaria cases in 2005, 2006, 2007, 2008 and 2009 were 8,618, 3,920, 1,819, 1,597 and 1,509 respectively (Table 2). Based on a population at risk of 65,000 people in the interior of Suriname, this leads to a decrease of estimated malaria incidence in the risk area from 13,258 in 2005 to 2,322 in 2009. The number of malaria notifications units increased and the surveillance system managed the inclusion of new MSDs into an updated national centralized malaria information system at BOG. The role of the fixed MSD clinic at Paramaribo as a diagnostic center for garimpeiros increased over the years since its establishment in 2007 (Table 2). Between 2005 and 2009 ten malaria outbreaks were detected in the interior of the country based on their respective threshold. ACDs performed in those communities resulted in a prevalence varying from 5.7% to 60.0%. All epidemics were controlled and malaria cases treated as specified in the national malaria treatment protocol.

**Table 2:** Overview of the number of malaria cases diagnosed in Suriname between 2005 and 2009 and of the number of malaria cases diagnosed at the fixed MSD clinic (Paramaribo) between 2007 and 2009

		2005	2006	2007	2008	2009
**No. of malaria cases diagnosed in Suriname**	Autochthonous malaria cases	8618	3920	1819	1597	1509

	Imported malaria cases	228	200	302	629	1140

**TOTAL**		**8846**	**4120**	**2121**	**2226**	**2649**

**No. of malaria cases diagnosed at the fixed MSD clinic (Paramaribo)**	Autochthonous malaria cases			122	248	292

	Imported malaria cases			149	515	960

**TOTAL**				**271**	**763**	**1252**

### Performance indicators

Table 1 shows the MM-MP indicator values by the end of the MM-MP. By the last reporting period (#23) most indicators substantially reached their targets, with the exception of the number of health facilities reporting no stock out of anti-malarials (3.6%), the number of people (19.2%) and communities (41.4%) reached by media awareness campaign and the number of re-impregnated nets (54.5%). The MM-MP scored B2 in the first four periods, B1 in period 5 and achieved A scores (A1, A2) from 2006 till early 2010; B1 was the final score during closure of the project in periods 22 and 23.

### Changes in the malaria situation

Malaria cases declined progressively after the introduction of ACT in 2004. The implementation of effective interventions by the MM-MP in 2005 achieved a decline of 82% of the total malaria cases in the country and 97% among communities living in the interior of the country under the coverage of Medical Mission Primary Health Care since 2000. The last malaria-related death was reported in 2007. The national API dropped significantly from 136 per 1000 population/year at risk to 24 per 1000 population/year at risk (*P *< 0.001). The remaining malaria risk areas in the country are concentrated along the border with French Guiana (France), specifically in mining areas. Most of the imported malaria cases find their origin here. Continued transmission in these areas, not targeted by the MM-MP, has led to the funding of a new malaria programme focused on decreasing malaria in the mining communities.

## Discussion and evaluation

In the last decade, malaria control efforts around the globe have gained a significant importance. The implementation of current anti-malarial interventions shows promising results in several countries [21]. In Suriname, we observed a progressive reduction in the number of malaria cases and deaths, especially after the scaling up of the interventions by the MM-MP starting in 2005. Today, malaria cases reported from the interior are almost all originating from gold mining areas and malaria transmission in the stabile village communities in the interior is almost non-existent. Many of the recent malaria patients in the country work in gold mining areas in French Guiana, but seek diagnosis and treatment in Suriname (imported malaria).

The onset of the decrease in malaria incidence started even before the implementation of the MM-MP and is thought to be due to the introduction of ACT as first line treatment for uncomplicated *P. falciparum *infections, which proved successful in Suriname and elsewhere [22,23]. To further eliminate any residual focus of malaria parasites, primaquine was also added to the ACT in 2007. Prompt diagnosis and treatment are still among the most effective interventions, as well as a continuous monitoring of drug resistance [8,24].

Suriname shifted from a conventional, mostly passive, malaria control strategy, to an active interaction with the populations at risk by introducing an integrated package of new (and old) means and methods for prevention and control. The focus is on reaching the risk-groups, even by using unprecedented means as the establishment of malaria notification points in remote areas through the training and supervision of local people.

Whether the decline in malaria in Suriname is due to the impact of the elaborate malaria control activities within the MM-MP, the use of effective drugs or other factors is subject of discussion. Between 2005 and April 2010 a significant decrease of *Anopheles *population densities in sentinel sites has been recorded by longitudinal vector surveillance studies. The very low density of *An. darlingi *mosquitoes from the sentinel sites after the excessive flooding of the major rivers in May 2006, suggests an impact of environmental factors on anopheline population densities. It is generally admitted that heavy rains can have an impact on mosquito densities by flooding the breeding sites and creating flood currents that carry away the immature mosquito stages [6,25,26]. On the other hand, reports exist on the mass killing of mosquitoes and significantly reduced indoor biting after the introduction of insecticide treated nets (ITNs) in malaria endemic areas [27,28]. This would support the hypothesis that the decrease of the (local) vector population density, and the ultimate disappearance of the vector from collections, may be a result of the mass distribution of LLINs and it may at the same time explain the lack of recovery of the mosquito populations after the floods.

The decrease of *An. darlingi *populations since the implementation of LLIN/IRS and the flooding in May 2006, led to a decrease in population densities and ultimately to a total absence of *An. darlingi *from collections in the sentinel sites. This partially explains the low yearly EIRs found in Jamaica and Drietabiki in 2006. Considering that flooding of rivers can have a (initial) negative impact on *Anopheles *populations [26,29], it is difficult to attribute the collapse of the *An. darlingi *populations entirely to either the introduction of LLINs or the floods, as it is likely due to the impact of both events. The vector populations did not recover during the following years. It could be hypothesized that the LLINs played a role in this by preventing the night-biting vector access to its preferred host. Whether or not the *An. darlingi *populations had disappeared or were simply below detection level is undetermined. It can be concluded, however that since the collapse of *An. darlingi *populations malaria transmission has virtually ceased to exist, which supports the observation of a sharp decline in autochthonous malaria transmission.

Even if the effect of the LLINs on *An. darlingi *remains unclear, the use of these nets is a rational choice in areas with anthropophilic and endophagic mosquitoes, like *An. darlingi*. Insecticide-treated nets are used in vector control programmes worldwide. Depending on the proportion of insecticide resistance in local vectors results vary, but generally the effects include reduced mosquito survival rates and sporozoite rates [27,30]. Successful control programmes with ITNs are found in Africa. The Gambia (West Africa), for instance, reported a 25% reduction in child mortality after a large-scale bed net impregnation campaign [31]. In Kenya, child mortality was reduced by 15-33% [32]. With the current low malaria burden and (locally) low density of malaria vector populations in Suriname the challenge will be to ensure a continued proper use of the bed nets by the people of the interior. The durability of the nets, including the impact of traditional washing methods on insecticide levels and integrity of the netting materials [33], needs to be studied in order to estimate at which time the nets should be replaced.

Other possible reasons for the reduction of malaria in Suriname need to be considered. Environmental factors (rainfall changes), changing human population movements (within Suriname and across borders) and increased awareness to fight malaria as a result of the media campaign might all have contributed to the decline of malaria incidence. Nevertheless, from the combined epidemiological and entomological data presented in this paper it can be concluded that the increased coverage of LLINs is probably among the main reasons of the substantial change in the malaria epidemiology profile in Suriname.

The historical successes of malaria control due to vector control are a motivation to have (again) an increased focus on the role of vectors in malaria transmission and on the opportunities for control and elimination. Considering the diversity of vector species, which can vary considerably in biting and resting behaviour, and the occurrence of insecticide resistance [27,34], the strategies for control worldwide move towards integrated vector management (IVM), combining the use of ITNs with other tools [35-38]. Success of a vector control strategy will depend on the appropriateness of control measures in a given situation. Knowledge of micro-epidemiology of malaria including ecology and behavior of the vector, social and cultural characteristics of the human population, and changes therein due to interventions or developments, should be guiding factors in deciding the course of action. By definition IVM is a decision-making process for the management of vector populations, so as to reduce or interrupt transmission of vector-borne diseases through the rational integration of all available measures. Suriname decided on combined use of LLINs, IRS and (re-)impregnation of nets. For the implementation of these measures the available health infrastructure, local personnel and non-governmental (support) groups were involved, and the activities were combined with disease control-related measures. IRS as one of the available vector control measures was discontinued after 2006 based on the rationale that mosquito populations had by that time collapsed. IRS can be a powerful tool in malaria control, provided that its impact on the mosquito populations is continuously monitored by entomological surveillance, and possible insecticide resistance being detected in time [38-40]. IRS used in combination with other malaria control measures, has led to significant decreases of malaria incidence in, for instance, tropical Asia and South America including neighboring Venezuela and Guyana [41]. Successes with DDT and pyrethroids varied over time in different countries, depending on the changes in biting behaviour and insecticide resistance of the vectors. The high costs of IRS programmes, as well as the varying successes ultimately led to deterioration of the programmes, which in turn led to resurgence of malaria in some countries (for instance in Sri Lanka; [42]). The re-introduction of IRS in Suriname by the MM-MP was based on high malaria incidence and high mosquito pressure in a specific malaria stratum of the country. Halting the IRS within the first year was a sensible decision, considering the decrease in mosquito biting intensity as well as in malaria incidence, the good acceptance of LLINs by the local population and the enormous logistic and financial resources involved in the execution of IRS.

In 2009, 76% (2032/2649) of all malaria cases diagnosed in the country (including imported malaria) were carried out by the fixed (47%) and mobile MSDs (29%). This means that malaria cases are no longer reported predominantly from the village communities in the interior, but almost all originate from gold mining areas. MM health centers have a wide area of coverage, but are often out of reach of gold miners. The gold miners, about 15,000 people [43], generally do not seek malaria treatment due to their illegal status and/or the high local transportation costs. Low accessibility to diagnosis and treatment for these gold miners have resulted in a flourishing black market of anti-malarial and other drugs, often of insufficient quality. Gold mining communities are currently the populations most at risk for malaria in Suriname. Improved access to health services and/or malaria services (free adequate diagnosis and high quality effective anti-malarial treatment) is necessary. The MM-MP introduced ACD in high risk areas, created new diagnostic points in mining areas and set up the fixed MSD clinic in Paramaribo, increasing the access to services, and thus decreasing the number of parasite carriers.

One of the most sensitive areas in terms of malaria control in Suriname is the eastern border region with French Guiana (France), which includes the Upper-Marowijne and Lawa rivers. This area has a high malaria incidence and a semi-mobile population, with many gold miners working on the French Guianese side of the border, but seeking supplies, equipment and health care in Suriname. France has a hard line policy towards illegal gold mining communities. This is thought to be the cause of the significant number of malaria cases originating from French Guianese gold mining areas, which are treated at the fixed MSD clinic in the capital of Suriname as these patients are not inclined to visit a health clinic in French Guiana.

The border area has been a focus for treatment and control efforts in both Suriname and French Guiana. Malaria control activities in Suriname led to a decrease of malaria in the French Guianese border region. Both countries recognize the need to come to unity in their approach of dealing with malaria. A cross-border initiative could be instrumental in preventing the re-introduction of malaria from French Guiana into Suriname.

Following the significant reduction of malaria in Suriname, national authorities evaluated the long-term goal; elimination. The MoH and the National Malaria Board decided in 2010 to develop a malaria control and elimination Plan 2011-2015 (MC&EP) [44]. The strategic vision is that the country will be malaria free by 2020 as a result of a full commitment of all stakeholders in further establishing and maintaining the RBM malaria control strategy. The most important strategic directions included in the MC&EP are improved malaria programme management and coordination, prompt and adequate case management, evidence-based IVM, continued and directional BCC/IEC, further improvement of the health system integration and its measurement and access. An important step towards the goal of elimination is the start-up of a new malaria control programme in 2009, managed by the MoH, which targets the high-risk group for malaria transmission, the (immigrant) gold miners. This programme provides these remote, ethnically diverse and mobile communities with easy access to malaria prevention, diagnosis and treatment.

The findings presented here support the hypothesis that financial investment in key effective interventions can have significant impact in reducing and even eliminating malaria in countries with low transmission.

## Conclusions

The success of the novel strategies for malaria control employed in the MM-MP in Suriname is evident through the significant reduction in the national malaria burden since their introduction in 2005. Malaria is reduced to pre-elimination levels in the stabile communities, even in previously high-risk areas. The communities considered most at risk nowadays are (mobile) gold miners, especially those working along the Suriname-French Guiana border. The challenge is to further reduce malaria using the available strategies as appropriate in the affected areas and populations. The target established by the Surinamese government to eliminate malaria in the country within a decade, requires a thorough understanding of transmission dynamics and a dedicated investment in key effective interventions. A bi-national approach towards controlling malaria along the border with French Guiana is necessary.

## Abbreviations

MM-MP: Medical Mission malaria programme; API: Annual parasitic index; ACT: Artemisinin-based combination therapy; GFATM: Global fund to fight aids tuberculosis and malaria; MM: Medical mission; BCC/IEC: Behavioral change communication/information education and communication; EIR: Entomological inoculation rate; IRS: Indoor residual spraying; LLIN: Long-lasting insecticide treated net; ITN: Insecticide-treated net; MSD: Malaria service deliverer; ACD: Active case detection; RDT: Rapid diagnostic test; BOG: Bureau of public health; M&E: Monitoring and evaluation; IVM: Integrated vector management; MoH: Ministry of health Suriname

## Competing interests

The authors declare that they have no competing interests.

## Authors' contributions

HH reviewed the available information on the history of malaria in Suriname, coordinated the entomological studies and monitoring and drafted the manuscript. LV reviewed the epidemiological databases, designed and adapted the strategies to the different strata, provided technical expertise and monitored the implementation of the interventions and co-drafted the manuscript. LH participated in the epidemiological studies and was responsible for the MM-MP programme data management. WT advised and critically revised the manuscript. All authors read and approved the final manuscript.
